# Within-host diversity of SARS-CoV-2 lineages and effect of vaccination

**DOI:** 10.21203/rs.3.rs-1927944/v1

**Published:** 2022-08-11

**Authors:** Haogao Gu, Ahmed Abdul Quadeer, Pavithra Krishnan, Daisy Y.M. Ng, Lydia D.J Chang, Gigi Y.Z. Liu, Samuel S.M. Cheng, Tommy T.Y. Lam, Malik Peiris, Matthew R. McKay, Leo L.M. Poon

**Affiliations:** 1 School of Public Health, LKS Faculty of Medicine, The University of Hong Kong, Hong Kong, China.; 2 Department of Electronic and Computer Engineering, The Hong Kong University of Science and Technology, Hong Kong SAR, China; 3 Centre for Immunology & Infection, Hong Kong Science and Technology Park, Hong Kong, China.; 4 Laboratory of Data Discovery for Health, Hong Kong Science and Technology Park, Hong Kong, China.; 5 HKU-Pasteur Research Pole, School of Public Health, LKS Faculty of Medicine, The University of Hong Kong, Hong Kong, China.; 6 Department of Electrical and Electronic Engineering, University of Melbourne, Parkville, VIC 3010, Australia; 7 Department of Microbiology and Immunology, The Peter Doherty Institute for Infection and Immunity, University of Melbourne, Melbourne, VIC 3000, Australia; 8 Department of Chemical and Biological Engineering, The Hong Kong University of Science and Technology, Hong Kong SAR, China

## Abstract

Viral and host factors can shape SARS-CoV-2 within-host viral diversity and virus evolution. However, little is known about lineage-specific and vaccination-specific mutations that occur within individuals. Here we analysed deep sequencing data from 2,146 SARS-CoV-2 samples with different viral lineages to describe the patterns of within-host diversity in different conditions, including vaccine-breakthrough infections. Variant of Concern (VOC) Alpha, Delta, and Omicron samples were found to have higher within-host nucleotide diversity while being under weaker purifying selection at full genome level compared to non-VOC SARS-CoV-2 viruses. Breakthrough Delta and Omicron infections in Comirnaty and CoronaVac vaccinated individuals appeared to have higher within-host purifying selection at the full-genome and/or Spike gene levels. Vaccine-induced antibody or T cell responses did not appear to have significant impact on within-host SARS-CoV-2 evolution. Our findings suggest that vaccination does not increase SARS-CoV-2 protein sequence space and may not facilitate emergence of more viral variants.

## Introduction

The SARS-CoV-2 pandemic continues to spread globally. Despite vaccination of over 60% of the world population^1^, the risk of SARS-CoV-2 reinfections and breakthrough infections is increasing due to the emergence of new viral variants^2,3^. Multiple variants of concern (VOC) have demonstrated the ability to evade naturally-acquired or vaccine-induced immunity^4-6^. Therefore, it is crucial to investigate the impact of vaccination on mutational and evolutionary processes of SARS-CoV-2.

Genomic surveillance has been used to trace the transmission and evolution of SARS-CoV-2 mutations at local, regional, and global scales throughout the pandemic^7-9^. However, there is still limited knowledge of how these mutations originate and accumulate within hosts. Within-host mutations can arise through replication errors or RNA damage/editing^10^ and they may be subject to fixation by stochastic (genetic drift) and deterministic (natural selection) processes. We and others have previously found that the SARS-CoV-2 transmission bottleneck between hosts is narrow^8,11-14^, suggesting that only few virions are transferred from the host during transmission. Most of the low-frequency mutations are not transmitted between patients, which constrains the use of intrahost single nucleotide variants (iSNVs) for effective contact tracing^12,15,16^. However, it remains important to investigate within-host diversity of SARS-CoV-2 to understand host-level evolutionary forces.

Studying SARS-CoV-2 within-host diversity under different conditions may reveal factors that control virus evolution. Host and viral factors can both contribute to within-host diversity. Host factors such as species (animals/humans)^17^, viral shedding time^18^, and immune status^19^ were previously reported to have effects on intrahost SARS-CoV-2 diversity. It was hypothesized that prolonged infections in hosts with distinct immunological backgrounds (e.g., animals or immunocompromised patients) may hasten viral evolution and lead to emergence of novel variants^17,20^. However, there is limited knowledge about post-vaccination characteristics of within-host selection pressures, which consistently act on the virus during the entire course of breakthrough infection. Besides, viral factors such as different virus lineages may also affect SARS-CoV-2 replication properties. SARS-CoV-2 VOCs have exhibited varying capacities to evade immunity^4,6^ and acquire higher transmissibility^21,22^. However, it is not clear whether different SARS-CoV-2 variants differ in within-host selection pressures.

To address these knowledge gaps, we analysed 2,146 deep sequenced SARS-CoV-2 samples collected in Hong Kong (HK) between mid-2020 and early 2022. The within-host diversity in SARS-CoV-2 infections from different lineages (VOCs: B.1.1.7 (Alpha), B.1.617.2 (Delta), B.1.1.529 (Omicron) and non-VOCs: B.1.36, B.1.36.27, and B.1.1.63) and in breakthrough (Delta or Omicron) infections after Comirnaty or CoronaVac vaccination were studied. Our results provide insights into the variation of within-host diversity, and the mutational patterns and potential selection pressures acting on viruses.

## Results

### Diversity of within-host mutations in SARS-CoV-2 samples

We analysed 2,146 SARS-CoV-2 samples from 2,053 different individuals, of whom 86 had multiple samples (totalling 2,053 representative samples and 93 repeated samples for technical control). The samples were collected from July-2020 to March-2022, covering the third to the fifth COVID-19 waves in HK. All samples had genome coverage ≥90% with >100 reads of sequencing depth (Supplementary Fig. 1) and with high viral loads (Ct value ≤25). The median sequencing depth ranged from 380 to 98,214 per sample. The samples belonged to major SARS-CoV-2 lineages including VOCs (B.1.1.7 (Alpha), B.1.617.2 (Delta), B.1.1.529 (Omicron, 20% are BA.1 and 80% are BA.2)) and non-VOCs (B.1.36, B.1.36.27, and B.1.1.63). The three non-VOC lineages were detected in the third (B.1.1.63) and fourth (B.1.36 and B.1.36.27) COVID-19 waves when no COVID-19 vaccine was available for use in HK^9^. For Delta and Omicron infections, samples from breakthrough infections after Comirnaty or CoronaVac (two-dose) vaccination were also included.

**Figure 1.**
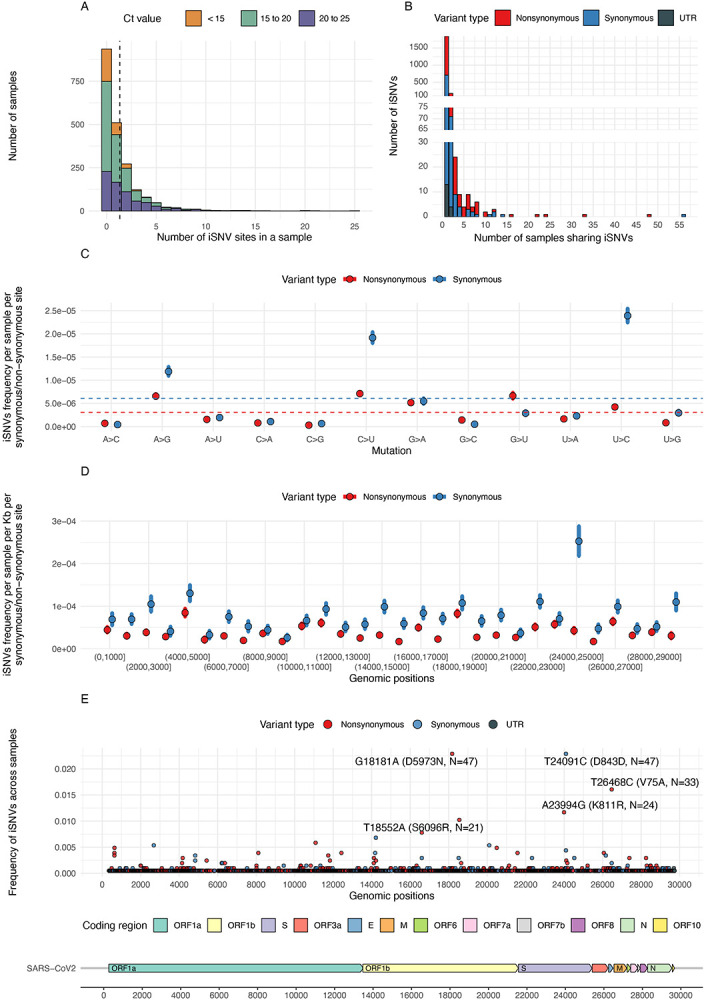
Statistics of within-host mutations in SARS-CoV-2 samples. **(A)** Distribution of number of iSNV site(s) in each sample, colored by ranges of Ct values. The dashed line shows the mean value of the distribution. **(B)** Distribution of number of sample(s) sharing iSNVs (e.g., if the iSNV identified in one sample was not shared with any other sample, then the number of samples sharing that iSNV equals to one (x = 1), and so on), colored by variant types. **(C)** Distribution of the frequency of iSNVs per sample per synonymous and per non-synonymous site (*d_S_* and *d_N_*) for different types of mutations, colored by variant types. The dashed lines show the average frequency of synonymous and non-synonymous iSNVs among all types of mutations. The points and error bars show mean and standard deviation values based on 10,000 bootstrap replicates at mutation level. **(D)** Distribution of the frequency of iSNVs per sample per Kb for synonymous and non-synonymous site (*d_S_* per Kb and *d_N_* per Kb) in different genomic regions of 1Kb length, colored by variant types. The points and error bars show mean and standard deviation values based on 10,000 bootstrap replicates at mutation level. **(E)** Distribution of high-frequency mutations shared by multiple samples, colored by variant types. Coding regions of the SARS-CoV-2 genome, based on the reference genome (GenBank: MN908947.3), are shown at the bottom of the figure.

For reliable analysis of within-host mutations, quality filtering steps were developed and validated using technical control samples (see Methods). We identified 2,731 iSNVs, with allele frequency between 5% to 50%, at 2,058 sites from 1,117 (54.4%) samples. Of these iSNVs, 1,694 (62.0%) of them were nonsynonymous, 1,016 (37.2%) were synonymous, and 21 (0.8%) were located in untranslated regions (mutations in the heading and tailing 100bp regions were excluded in this study). We did not identify detectable iSNVs in the other 936 (45.6%) samples. Overall, the mean number of iSNVs per sample was 1.33 (Fig. 1A, dashed line). This iSNV detection rate is similar to a previously reported level^11,16^, but lower than some other studies^15,23^, presumably due to differences in variant filtering criteria. Of the iSNV sites, 1,801 (88%) were uniquely observed in a single patient sample. This suggests that most iSNVs are sporadic mutations occurring at distinct positions rather than recurrent mutations occurring at specific mutation hotspots (Fig. 1B).

We found a weak correlation between viral load (Ct value) and the number of iSNVs per Kb. Samples with higher viral load (lower Ct value) generally had less iSNVs (Fig. 1A and Supplementary Fig. 2A). However, while viral load decreased with detection lag (time since symptom onset) (Supplementary Fig. 2B), the correlation between detection lag and number of iSNVs was not found to be significant (Supplementary Fig. 2C). We also found that the viral load did not significantly correlate with minor allele frequency (MAF) (Supplementary Fig. 2D). Consistent with other studies, these results (Supplementary Fig. 2A-2D) suggest that enrichment of iSNVs negatively correlates with viral load^11,12,15,16^, but varies less with time from symptom onset^16^. To avoid artefacts due to low viral load^16^, we only included samples with a Ct value ≤25 and adjusted the number of iSNVs per Kb (referred to as incidence of iSNVs hereafter) by linear regression functions (see Methods) in the downstream comparative analysis. With these adjustments, the correlation of Ct value and number of iSNVs per Kb became insignificant (Supplementary Fig. 2E). We found the mean number of iSNVs per Kb to be 0.045 across the full genome and the highest incidence of iSNVs were found in the ORF8 and ORF7a genes (Supplementary Table 1).

Consistent with previous reports^23,24^, we found some mutation types (C→U, G→A, A→G, U→C, and G→U) occurred with higher-than-average frequencies, measured in terms of the number of synonymous/nonsynonymous iSNVs per synonymous/nonsynonymous site (i.e., *d_S_* and *d_N_*) (Fig. 1C; points above the dashed lines). The high frequency of C→U/G→A and A→G/U→C mutants support the hypothesis of RNA editing *in vivo* via APOlipoprotein B Editing Complex (APOBEC) and Adenosine Deaminase Acting on RNA (ADAR) enzymes^10,25^, respectively. Interestingly, we observed a higher mutation frequency of G→U, but a lower mutation frequency of C→A, which suggests a strand bias of the G→U mutation. The G→U mutation may be associated to Reactive Oxygen Species (ROS)-related processes^26^. In different regions of the SARS-CoV-2 genome, we observed uneven *d_S_* and *d_N_* (P<0.001, Kruskal-Wallis rank sum test among regions with length of 1Kb) and the highest frequency was found in *d_S_* in the Spike gene region (genomic position from 24000 to 25000 in Fig. 1D). For all regions, the number of synonymous iSNVs per Kb per synonymous site (*d_S_* per Kb) are higher than the average number of nonsynonymous iSNVs per Kb per non-synonymous site (*d_N_* per Kb) (Fig. 1D). There were some shared iSNVs, i.e., found in multiple samples from different patients, with five of them (labelled in Fig. 1E) observed in more than 20 samples (frequency>1%). These five high-frequent iSNVs were found in samples from more than one SARS-CoV-2 lineage and under different vaccination statuses, suggesting mutation homoplasy rather than shared mutations from direct transmissions (Supplementary Table 2).

### VOC samples exhibit higher within-host diversity but weaker purifying selection than non-VOC samples

To study the within-host diversity between different groups, i.e., SARS-CoV-2 lineages for different vaccination statuses, we calculated the incidence of iSNVs (adjusted number of iSNVs per Kb), abundance of iSNVs (MAF for iSNVs), and nucleotide diversity (*π*, average number of nucleotide differences per site between pairwise reads)^27^ for samples within each group (see Methods). Combinational use of the three complementary indices can help illustrate viral mutant spectrum dynamics^28^. Essentially, incidence of iSNVs correspond to counts of mutational sites in a sample (the breadth of the mutant spectrum), abundance of iSNVs reflects the mutational frequency of each site in the sample (the height/intensity of the mutant spectrum), and nucleotide diversity (*π*) is a functional index based on the total pairwise difference among observed haplotypes (the degree of polymorphism of iSNVs within a sample). Nucleotide diversity (*π*) can be further characterized as synonymous and nonsynonymous nucleotide diversity (*π_S_* and *π_N_*) in coding regions. In general, excess nonsynonymous polymorphism (*π_N_* > *π_S_*) points to diversifying/positive selection while excess synonymous polymorphism (*π_N_* < *π_S_*) indicates purifying selection. Relatively weak selection forces are observed when stochastic changes (genetic drift) dominate (*π_N_* ≈ *π_S_*)^29^.

**Figure 2.**
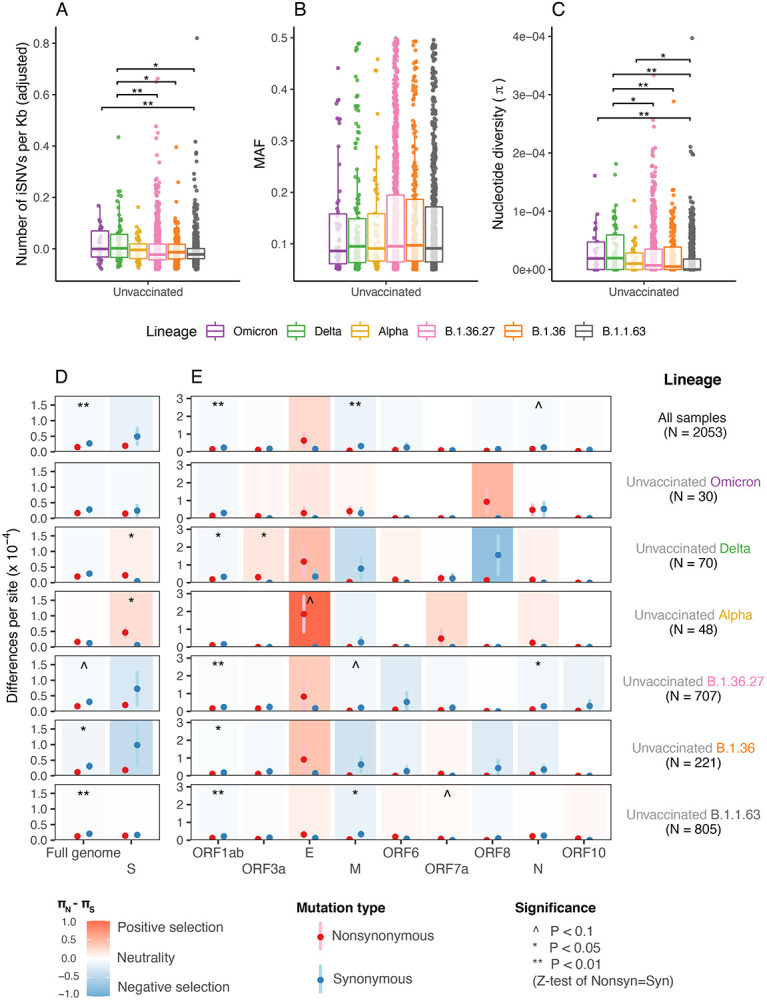
Comparison of within-host mutation profiles between unvaccinated VOC and unvaccinated non-VOC samples. **(A-C)** Full genome incidence of iSNVs (adjusted number of iSNVs per Kb), abundance of iSNVs (minor allele frequencies, MAF), and nucleotide diversity (π) of different samples. For all box plots, the bold horizontal line inside the box shows the median, the upper and lower edges of the box indicate the first and the third quartiles, and whiskers extend to span a 1.5 interquartile range from the edges. Pairwise comparisons between groups were performed by two-sided two-sample Wilcoxon tests; the pairs with P-value ≤ 0.01 and ≤ 0.05 are labelled with “**” and “*” respectively. **(D-E)** Full-genome and gene-specific within-host nonsynonymous nucleotide diversity (*π_N_*) and synonymous nucleotide diversity (*π_S_*) in samples from different groups. The points and error bars show the mean and standard deviation values under 10,000 bootstrap replicates at codon level. Significance was evaluated using Z-tests of the null hypothesis that *π_N_* – *π_S_* = 0 (10,000 bootstrap replicates, codon unit); P-value ≤ 0.01, ≤ 0.05 and ≤ 0.10 are labelled with “**”, “*” and “^”, respectively.

Lineage-specific effects on iSNVs can be characterized by comparing unvaccinated samples between lineages. We found Delta samples without vaccination (designated “unvaccinated Delta samples”) had higher incidence of iSNVs than samples from the non-VOC lineages (medians: 0.002 for Delta vs. −0.022, −0.014 and −0.023 for B.1.1.63, B.1.36 and B.1.36.27 respectively, P<0.05; Fig. 2A and Supplementary Table 3). The unvaccinated Omicron samples also had higher incidence of iSNVs than unvaccinated B.1.1.63 samples. Notably, the median incidence of iSNVs for VOC lineages (Alpha, Delta and Omicron) are all higher than non-VOC lineages (Fig. 2A), suggesting different genetic backgrounds of viruses had different within-host mutation rates. No significant difference was observed between abundance of iSNVs across unvaccinated VOC and non-VOC samples (Fig. 2B).

Similar to what was observed for the incidence of iSNVs, the nucleotide diversity in Delta samples was significantly higher than for samples from all three non-VOC lineages (Fig. 2C and Supplementary Table 3). The overall nucleotide diversity for unvaccinated Omicron and Alpha samples were statistically significantly higher than samples from the third local wave lineage B.1.1.63 samples (P<0.05, Fig. 2C and Supplementary Table 3). Overall, unvaccinated VOC samples had higher median nucleotide diversity compared to the non-VOC samples (Fig. 2C), suggesting infection with VOCs may induce greater within-host genetic variation.

We found evidence of significant purifying selection in the SARS-CoV-2 genome (top row, Full genome column in Fig. 2D) and most samples have excess synonymous polymorphisms (*π_N_* < *π_S_*, P<0.001 by two-sided Wilcoxon rank sum test). The mean value of *π_N_* – *π_S_* is −1.18 × 10^−5^ (*π_N_/π_S_* = 0.56) across the full genome for all samples, which is consistent with previous reports^11^ (*π_N_/π_S_* = 0.55), but differs from what was observed in other mammalian samples^17^.

For the unvaccinated non-VOC (B.1.1.63, B.1.36 and B.1.36.27) samples, purifying selection was observed at the full-genome level (Full genome panel in Fig. 2D). By contrast, all three unvaccinated VOC (Alpha, Delta and Omicron) samples had overall unbiased selection (*π_N_* ≈ *π_S_*) at the full genome level which is statistically indistinguishable from neutrality. At the individual gene level, evidence for positive selection was observed for the Spike gene in both unvaccinated Alpha and Delta samples (*π_N_* – *π_S_* = 4.05 × 10^−5^ and 1.87 × 10^−5^, column S in Fig. 2D and Supplementary Table 4). However, unvaccinated non-VOC samples generally showed neutral to purifying selection in the Spike gene (*π_N_* – *π_S_* = −8.00 × 10^−5^, −5.21 × 10^−5^ and −0.25 × 10^−5^). This result suggests that, compared to viruses from non-VOCs lineages, those from VOC lineages are under less purifying selection pressure at the within-host level.

For other coding regions, our data suggests little evidence of lineage-specific changes in selection pressure. Neutral or purifying selection was generally observed. For example, in ORF1ab, in all cases the synonymous nucleotide diversity is higher than or similar to the non-synonymous nucleotide diversity (Fig. 2E, ORF1ab column). Possible positive selection was observed in ORF3a in unvaccinated Delta samples (P<0.05, Fig. 2E), E in unvaccinated Alpha samples (P<0.1, Fig. 2E), and ORF7a in unvaccinated B.1.1.63 samples (P<0.1, Fig. 2E).

### Vaccination appears to increase the within-host mutation rate and purifying selection pressure on VOC samples

**Figure 3.**
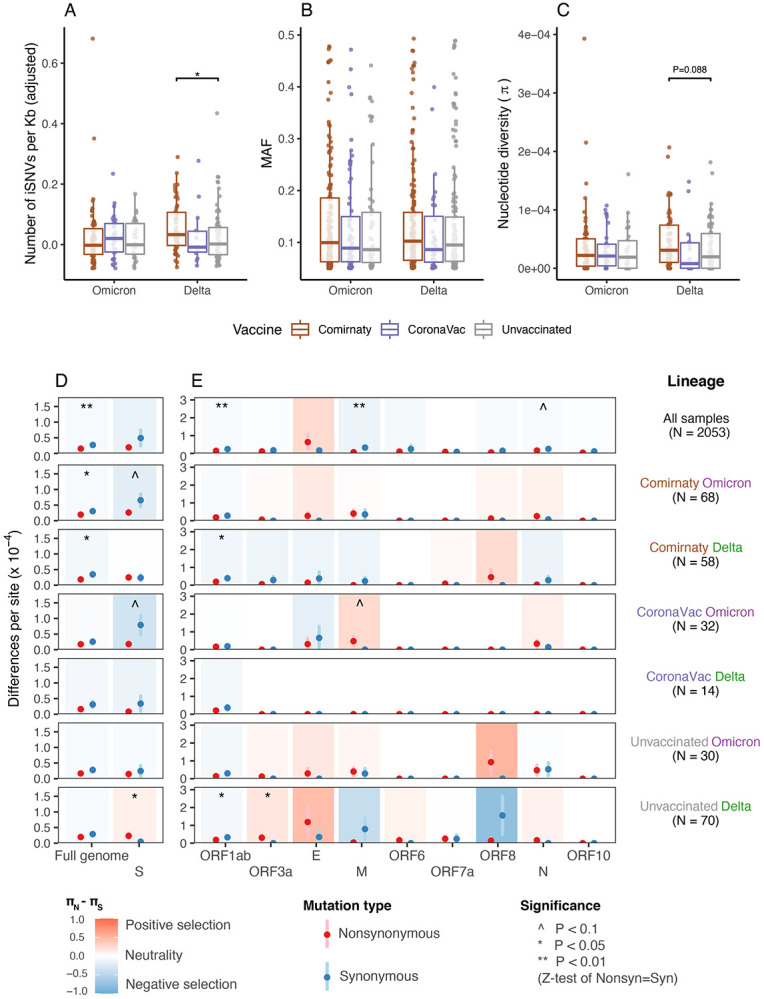
Comparison of within-host mutation profiles between vaccinated and unvaccinated Delta and Omicron samples. **(A-C)** Full-genome incidence of iSNVs (adjusted number of iSNVs per Kb), abundance of iSNVs (minor allele frequencies, MAF) and nucleotide diversity (π) of different samples. For all box plots, the bold horizontal line inside the box shows the median, the upper and lower edges of the box indicate the first and the third quartiles, and whiskers extend to span a 1.5 interquartile range from the edges. Pairwise comparisons between groups were performed by the two-sided two-sample Wilcoxon test; the pairs with P-value ≤ 0.01 and ≤ 0.05 are labelled with “**” and “*” respectively. **(D-E)** Full-genome and gene-specific within-host nonsynonymous nucleotide diversity (*π_N_*) and synonymous nucleotide diversity (*π_S_*) in samples from different groups. The points and error bars showed the mean and standard deviation values under 10,000 bootstrap replicates at codon level. Significance was evaluated using Z-tests of the null hypothesis that *π_N_* – *π_S_* = 0 (10,000 bootstrap replicates, codon unit); P-value ≤ 0.01, ≤ 0.05 and ≤ 0.10 were labelled with “**”, “*” and “^”, respectively.

The incidence of iSNVs and nucleotide diversity may also be affected by vaccination. By studying the samples of breakthrough infections from fully vaccinated (with two-doses of Comirnaty or CoronaVac vaccines) patients, we found that the incidence of iSNVs in Comirnaty Delta virus samples was significantly higher than that from the unvaccinated Delta samples (Fig. 3A) and the Comirnaty Omicron samples (Supplementary Fig. 3A). Within Delta samples, higher incidence of iSNVs in Comirnaty samples compared to unvaccinated samples suggests vaccine-specific effects on within-host mutation rate. However, a similar effect was not observed for Omicron samples (Fig. 3A). One possible explanation for the difference between Delta and Omicron samples could be the waning of vaccine effectiveness, as overall a longer time had passed since receiving the second dose for Omicron-infected vaccinated patients in our data (Supplementary Fig. 4). It is also possible that different levels of immune evasion between Omicron and Delta infections may play a role, since neutralizing antibody titers induced by the Comirnaty vaccine against Omicron were lower than those against Delta^30^. Unlike incidence of iSNVs (Fig. 3A), abundance of iSNVs was similar across vaccinated and unvaccinated samples (Fig. 3B), while nucleotide diversities (*π*) were only marginally significantly higher (P<0.1) in Comirnaty Delta samples compared to unvaccinated samples (Fig. 3C), suggesting that the overall level of genetic variation was not markedly increased by vaccination.

We found elevated purifying selection pressure at the full-genome level from Comirnaty vaccination, where significant *π_N_* < *π_S_* were observed in vaccinated samples but not for unvaccinated samples (Fig. 3D, Full genome column). The enhanced purifying selection in Comirnaty Delta and Omicron samples was mainly contributed by increased *π_S_* (Supplementary Table 4). At the Spike gene level, the significant positive selection on the unvaccinated Delta samples was not observed in vaccinated Delta samples (*π_N_* – *π_S_* = 1.87 × 10^−5^, 0.09 × 10^−5^ and −2.58 × 10^−5^ for unvaccinated Delta, Comirnaty Delta and CoronaVac Delta, respectively; column S in Fig. 3D, and Supplementary Table 4). While the selection pressure on the Spike gene in unvaccinated Omicron samples (*π_N_* – *π_S_* = −0.97 × 10^−5^) was not significantly different from neutrality, the purifying selection was moderately significant in those with Comirnaty or CoronaVac vaccination (*π_N_* – *π_S_* = −3.94 × 10^−5^ and −6.11 × 10^−5^ for Comirnaty and CoronaVac, respectively, P<0.1). Collectively, Comirnaty vaccination may increase synonymous nucleotide diversity and thereby purifying selection pressures on Delta and Omicron viruses at the full genome level. For the Spike gene, the observed positive/neutral selection pressures acting on Delta and Omicron samples could be shifted to neutral/purifying selection in those with CoronaVac or Comirnaty vaccination. Similar to the lineage-specific results, we did not find consistent vaccination-specific changes in selection pressure for other coding regions. Neutral or purifying selection was predominant (Fig. 3E), with possible positive selection observed in the M gene in CoronaVac Omicron samples (P<0.1).

Positive selection in coding regions of VOC-specific and vaccination-specific samples (Fig. 2E and 3E) suggests diversifying mutations that can potentially lead to higher chance of phenotypic changes. To identify putative hotspot regions with excessive positive selection, we analysed sliding windows (size of 30 codons) across each protein-coding region. Consistent to the results above, we found most genomic regions were under purifying selection. Seven candidate targets of positive selection were found in ORF1ab, ORF7a and E (Supplementary Fig. 5 and Supplementary Table 5). Of these, three regions (nsp3:448-451, nsp15:278-279 and E:40-47) had partial overlap with the regions determined to be under positive selection in an independent study^24^.

### No significant selection on within-host mutations from immune pressure

To investigate whether the within-host mutations detected in our vaccinated samples enable immune escape, we studied the overlaps of identified within-host mutations with known neutralizing antibody (nAb) escape mutations in the Spike gene and with experimentally-determined T cell epitopes across the full genome.

**Figure 4.**
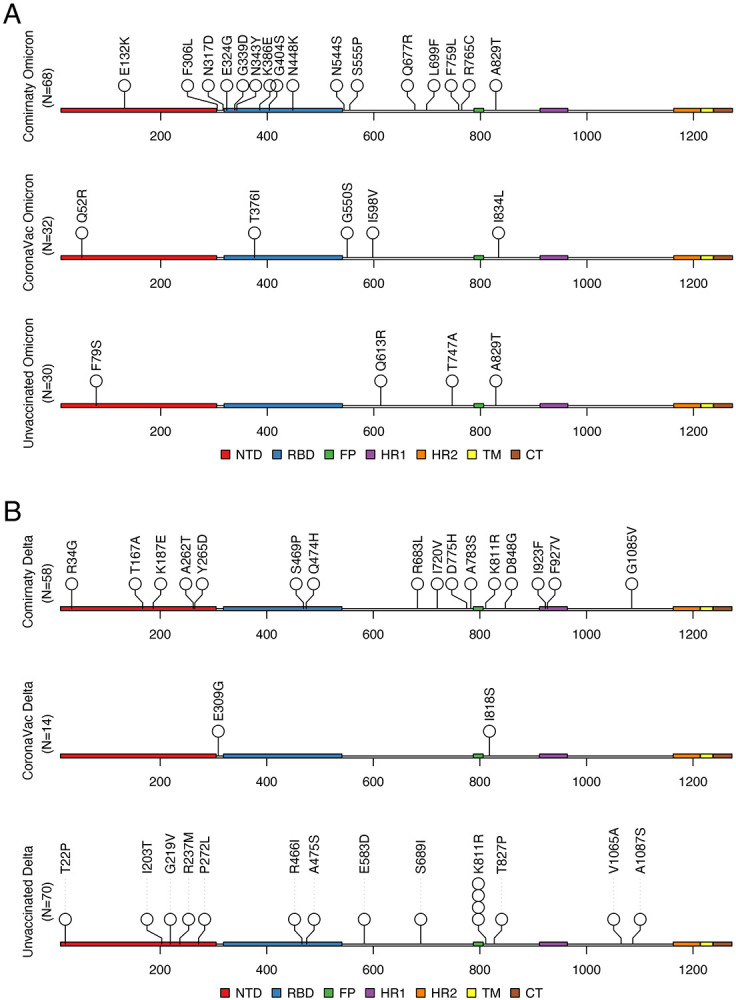
Spike mutations identified in unvaccinated and vaccinated Delta and Omicron samples. Each circle represents one mutation identified in one sample in this study. **(A)** Identified within-host mutations in Omicron samples; **(B)** Identified within-host mutations in Delta samples.

Although we found mutations on the receptor-binding domain (RBD) and near the S1/S2 cleavage site (e.g., R683L), the overall mutations did not significantly cluster in any specific regions of the Spike gene (Fig. 4A, 4B). The total number of RBD mutations seem to be higher in Comirnaty Omicron samples (6 RBD mutations in 68 Comirnaty Omicron samples vs. zero mutation in 30 unvaccinated Omicron samples, Fig. 4A), however this difference was not significant (P=0.25, Chi-squared Test). Except for the K386E and N448K mutations found in two different Comirnaty Omicron samples (Fig. 4A), which may have mild effects on antibody escape (Supplementary Fig. 6A), the other identified mutations in the RBD region in all vaccinated Omicron and Delta samples were not on key antigenic sites (Supplementary Fig. 6A and 6B). For the NTD region, except for the A262T mutation found in one Comirnaty Delta sample, none of the other within-host mutations overlapped with the known NTD antigenic supersite^31^ or with mutations that have been reported to affect neutralization of NTD-targeting nAbs^32,33^.

**Figure 5.**
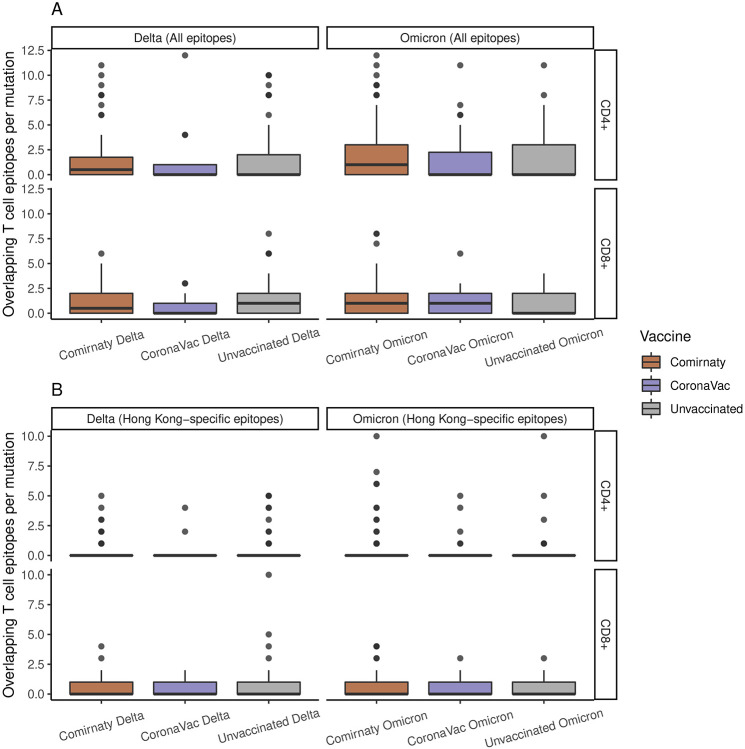
Overlapping known SARS-CoV-2 CD8+ and CD4+ T cell epitopes per mutation in unvaccinated and vaccinated Delta and Omicron samples. **(A)** Analysis based on all known SARS-CoV-2 T cell epitopes. **(B)** Analysis based on T cell epitopes associated with HLA alleles prevalent in the Hong Kong population. Pairwise comparisons within groups were performed by the two-sided two-sample Wilcoxon test. For all box plots, the bold horizontal line inside the box shows the median, the upper and lower edges of the box indicate the first and the third quartiles, and whiskers extend to span a 1.5 interquartile range from the edges.

In addition to nAbs escape mutations, T cell escape mutants have been shown to be selected under immune pressure in infections from influenza viruses^34,35^. However, the relationship between within-host mutations and T cell responses induced by SARS-CoV-2 infection or vaccination remains largely unknown. To investigate whether the variation in samples from breakthrough infections are related to host T cell responses, we studied the overlap between within-host mutations (minor allele variants) and known T cell epitopes. A total of 1324 CD8^+^-specific and 961 CD4^+^-specific T cell epitope-HLA (human leukocyte antigen) pairs were compiled (Methods). The distributions of these epitope-HLA pairs across SARS-CoV-2 proteins and across HLAs are shown in Supplementary Fig. 7. Considering T cell epitopes across all proteins, the average number of overlapping CD8^+^ and CD4^+^ epitopes per mutation was generally similar between different groups (Supplementary Fig. 8). Focusing on vaccinated and unvaccinated samples, we observed no significant difference in the number of overlapping epitopes per mutation (Fig. 5A), which is suggestive of no T cell-based selection on within-host viral evolution. When limited to iSNVs within the Spike gene, a marginally higher number of overlapping CD4^+^ T cell epitopes was found in Comirnaty Omicron samples compared to unvaccinated Omicron samples, but the difference was not significant (P=0.09, Supplementary Fig. 9A).

Since the samples were sequenced from HK cases, we repeated the above analysis while focusing on the epitopes associated with HLAs prevalent in the HK population (Supplementary Fig. 10, Methods). As for the above results (Fig. 5A and Supplementary Fig. 9A), we did not observe a significant difference in the number of overlapping CD8^+^ and CD4^+^ T cell epitopes per mutation between the vaccinated and unvaccinated samples in the full genome (Fig. 5B) or in the Spike gene (Supplementary Fig. 9B).

While the two candidate regions of positive selection mentioned in the previous section (nsp3:448-451 and E:40-47) overlapped with many CD8^+^ T cell epitopes (N=8 and N=5), these associations did not reach statistical significance (Supplementary Table 5). Overall, we did not identify a surge of antibody escape mutations in any group, and different groups had a similar level of mutation rates in T cell epitope regions.

## Discussion

In this study we have analysed Illumina amplicon data from 2,146 SARS-CoV-2 samples to estimate intra-host variation of SARS-CoV-2 under different conditions. Similar to earlier studies, we show that incidence of iSNVs in SARS-CoV-2 samples is low (0 to 2 iSNVs per sample)^11,16^ and that sample viral loads negatively correlate with within-host mutation rates^11,12,15,16^, which suggests low viral load specimens are prone to bias toward falsely high iSNVs rates. In agreement with reports from Tonkin-Hill et al.^15^ where SARS-CoV-2 samples with lower Ct value show good concordance in allele frequencies between replicates, we also found the cut-off of Ct ≤25 can avoid most outliers. Evidence of RNA editing at the full genome level, e.g., the widely reported biased C→U/G→A and A→G/U→C pairs of mutations^23,24^, was observed in our study. We also found strong strand asymmetry of G→U mutations in our data, suggestive of RNA damage or RNA editing (rather than replication errors) on the plus stand^15^ and possible association with ROS-related processes^26^. The frequency of synonymous mutations is higher than expected (*d_N_* < *d_S_*), corresponding to overall purifying selection on within-host mutations of SARS-CoV-2. Collectively, the general within-host virus sequence diversity in the samples from HK was similar to samples from other geographical areas collected at different timepoints^11,16^.

Different lineages of SARS-CoV-2 have different properties, including different levels of transmissibility^21,22^, disease severity^36,37^, viral load^37,38^, tissue affinity^39^, ability of vaccine breakthrough^4-6^, etc. Here, we found SARS-CoV-2 VOC Delta, Omicron and Alpha samples had higher within-host mutation rate and/or nucleotide diversity than non-VOC lineages. Such increased mutation rate is independent of viral load, suggesting different intrinsic biological properties between variants may play a role. As the entire infected population in HK by the end of 2021 was <0.2%, our observation is unlikely affected by interference induced by prior natural infection. Various mutations have been shown to account for different viral properties, e.g., ACE2 binding (e.g., K417N, N501Y)^40^, and immune escape (e.g., T478K, L452R)^41^. The increased nonsynonymous nucleotide diversity and putative diversifying selection in VOC samples (Supplementary Table 4) suggest that VOC viruses have a greater capacity to explore protein sequence space and therefore are more likely to incur a fitness change. This result is in line with VOCs’ ability to spread and result in multiple sub-lineages, and warrants close monitoring of their molecular evolution in the future.

Vaccination is another factor which may affect the within-host evolution of the virus. We studied samples from Comirnaty and CoronaVac vaccine breakthrough infections and found that vaccination may be associated with increased mutation rates and increased purifying selection. We found Comirnaty vaccination may be associated with increased within-host mutation rate and nucleotide diversity in SARS-CoV-2 Delta-variant samples. Notably, the increased nucleotide diversity in specimens of Delta breakthrough infection in Comirnaty vaccinated individuals is mostly synonymous rather than non-synonymous (*π_S_* = 3.47 and 2.90 for Comirnaty Delta and unvaccinated Delta samples respectively, Supplementary Table 4). We found Comirnaty vaccination increased synonymous nucleotide diversity and thus purifying selection pressure at both the full genome and Spike gene levels, while CoronaVac vaccination showed similar effects only at the Spike gene level. It has been reported that Comirnaty vaccine is markedly more immunogenic than CoronaVac vaccine and this may contribute to our observation^42^. It is also relevant to note that Comirnaty vaccine only has the Spike protein as an immunogen but appears to impact on purifying selection elsewhere in the genome. This may be a result of greater suppression of viral replication. Crucially, additional purifying selection pressures imposed by vaccination may limit the evolutionary protein sequence space as non-synonymous nucleotide diversity does not seem to be increasing. Overall, Comirnaty and CoronaVac vaccination seemingly amplifies the within-host purifying selection in VOCs.

We did not observe enrichment of VOC defining mutations for the non-VOC samples (data not shown), which suggests that convergent evolution of VOC mutations is infrequent. Only three of the mutations observed in our vaccinated samples overlap with known nAb escape supersites on the Spike NTD or RBD regions and the predicted RBD immune escape potential is only mildly (less than 10% immune escape) affected by these mutations. Evolution of T cell epitopes under selection by the host immune system has been reported for other viruses^43-45^, and T cell responses to SARS-CoV-2 have also been reported in most COVID-19 patients^46^. However, we did not detect significant vaccination-specific T cell pressure on within-host diversity, suggesting vaccine-induced pressure may not enhance exploration of immune escape pathways.

As HK used an elimination strategy to control COVID-19, the individuals investigated in our study can be reliably categorised as immunologically naïve or vaccinated individuals, which is a significant advantage of our study. Nonetheless, our study has some limitations. The sample size for some groups in this study is small due to limited availability of samples. Although the vaccinated and unvaccinated samples in this study were collected at similar time points after symptom onset (P=0.801, two-sided Wilcoxon rank sum test), most of the studied cases have only single time point samples, and we lack serial samples data of breakthrough infections for studying the temporal changes of within-host selection pressures. In studying the effect of T cell pressure on within-host viral evolution, we could not perform an individual-based analysis since HLA typing of the patients was not performed. As most of the individuals in our study were either infection naïve or vaccinated prior to infection, the effect of hybrid immunity on SARS-CoV-2 within-host evolution could not be addressed and requires further investigation.

In conclusion, our work suggests that SARS-CoV-2 within-host evolution may exhibit different patterns in different virus lineages and in vaccinated individuals. We found that Comirnaty and CoronaVac COVID-19 vaccination increases within-host purifying selection in VOCs, providing evidence that vaccination may limit the exploration of protein sequence space and emergence of more viral variants.

## Methods

### Samples and sequencing

This study was conducted under ethical approval from the Institutional Review Board of the University of Hong Kong (UW 20-168). We included Illumina amplicon data from 2,053 samples from lineages B.1.1.7 (Alpha), B.1.617.2 (Delta), B.1.1.529 (Omicron) and B.1.1.63/B.1.36/B.1.36.27 (variants in the third and fourth local wave) collected from 2020-07-04 to 2022-03-01 in HK. All the samples were from patients who were either unvaccinated or fully vaccinated (received two doses of vaccines) with Comirnaty or CoronaVac vaccines. The number of samples included in the analysis are presented in Supplementary Table 6. The metadata and vaccination records of RT-PCR confirmed cases of COVID-19 were collected from the public data released by HK government since July 29, 2021 (https://gia.info.gov.hk/general/202107/29/P2021072900356_373472_1_1627542548101.pdf).

To obtain high quality sequence results, we only included samples with a cycle threshold (Ct) value ≤25 and with sufficient genome coverage and sequencing depth (sequencing depth ≥100 properly paired reads are required at >=27000 genomic sites for every sample) after Illumina sequencing. RNA samples were sent to a World Health Organization reference laboratory at the University of HK for full-genome analyses (Institutional Review Board no. UW 20–168). Virus genome was reverse transcribed with multiple gene-specific primers targeting different regions of the viral genome. The synthesized cDNA was then subjected to multiple overlapping 2-kb PCRs for full-genome amplification. PCR amplicons obtained from the same specimen were pooled and sequenced by using the Novaseq or iSeq sequencing platform (Illumina). Sequencing library was prepared by using Nextera XT (Illumina). Generated sequencing reads were quality trimmed by fastp with parameters (“-q 30 -5 -3 -c --detect_adapter_for_pe -l 50”). Potential PCR duplicates were removed by samtools markdup (v1.11). The trimmed reads were mapped to a reference virus genome by using BWA-MEM2 (v2.0pre2), and genome consensus was generated by using iVar (v1.3.1) with the PCR primer trimming protocol (minimum sequence depth of 100 and minimum Qvalue of 30).

### Variant calling and quality control

The consensus-level single nucleotide polymorphisms (SNPs) and intrahost single nucleotide variants (iSNVs) were called by iVar variants (v1.3.1) with reference to the Wuhan-Hu-01 sequence. To limit the analysis to high quality SNPs and iSNVs, the following filtering criteria were applied:

1. SNVs were called from samtools mpileup files from quality-filtered reads alignment bam files using pysamstats.
2. After filtering based on MAF threshold of 0.05, we identified 24,161 iSNVs in 2,051 samples.
3. After filtering for iSNVs with strong strand bias (we kept iSNVs with strand ratio < 1/10), we identified 5,949 iSNVs in 1,643 samples.
4. After filtering for serial adjacent disjoint mutations (≥3 mutations within 30 nucleotides sliding window, likely relating to sequencing errors), we identified 5,808 iSNVs in 1,642 samples.
5. After filtering for iSNVs by minimum depth of 100 reads, we identified 5,073 iSNVs in 1,587 samples.
6. After filtering heading/tailing 100bp UTR region, binding regions of PCR primers, and previously known problematic sites^47^, we identified 3,439 iSNVs in 1,129 samples.
7. Finally, removing samples with possible co-infection/contamination, we identified 2,731 iSNVs in 1,117 samples.

To further validate that the identified iSNVs are of high confidence and are reproducible, we tested another 93 technical control samples from 86 cases sequenced by different sequencing runs and platforms. Using the same filtering criteria, we found iSNVs are significantly more reproducible among technical control samples from the same patient. Specifically, for the technical control samples with at least one iSNV, 54.2% (median) of the iSNVs were reproducible between samples from the same patient, compared to 0% (median) of the iSNVs being reproducible among samples from different patients (P<0.001, two-sided Wilcoxon rank sum test).

### Mutation summary statistics

#### Incidence of iSNVs and minor allele frequency

The incidence of iSNVs (number of iSNVs per Kb) was calculated by dividing the number of iSNVs with the number of genomic positions with sufficient coverage of reads (sequencing depth ≥ 100). The adjusted incidence of iSNVs is the residual (that is response minus fitted values) calculated by least-squares linear model (“lm” function in R 4.1.0) with the numbers of iSNVs per Kb (response variable) and Ct values (explanatory variable) from all the studied samples. The minor allele frequency (MAF), representing the abundance of iSNVs, was calculated directly from the alignment mpileup files using pysamstats (v1.1.2).

#### Nucleotide diversity (*π*)

Nucleotide diversity (*π*) is a summary metric of the degree of polymorphism of iSNVs within a sample and is tolerant of biases from sequencing depth^48^. We use it to measure the degree of iSNVs polymorphism within a sample. For every sample, where *n_i_* sequences (NGS reads) of nucleotide *i* are observed, nucleotide diversity (*π*) can be calculated based on pairwise difference between sequencing reads. as

$$\pi =\frac{\sum_{i\neq j}n_{i}n_{j}}{\frac{1}{2}N(N-1)},$$

where N is the total number of sequences.

### Selection analysis

The nucleotide diversity can be separately calculated for synonymous (*π_S_*) and non-synonymous changes (*π_N_*) in coding regions. We calculated the *π_N_* and *π_S_* in this study using SNPGenie^49^ with self-curated input vcf files based on the above identified iSNVs. For hypothesis testing of selection neutrality (*π_N_* = *π_S_*), Z-tests using a bootstrap method (codon unit, 10,000 replicates for genes and sliding windows) was applied. The scripts of sliding window analysis for positive selection are largely based on a previous analysis developed by the author of the software (https://github.com/krisp-kwazulu-natal/within-host-diversity-manuscript-analysis-code/blob/a276286680de3723e2b1e70f7a060750892cf8af/scripts/diversity_selection_analyses.R). The usage of this software in our study was approved by the author. Sliding windows of thirty codons and step size of one codon were used because this did not exceed the length of ORF10 (thirty-nine codons).

### Neutralizing antibody escape mutations

The Spike RBD mutations found in all Omicron and Delta samples were analyzed separately with the Escape Calculator for SARS-CoV-2 RBD^50^. The calculations are based on deep mutational scanning of a large set of RBD targeting antibodies which are known to neutralize the ancestral Wuhan-Hu-1 strain. The mutation escape strength in the Escape Calculator was set to the default value of 2.

For NTD, an antigenic supersite has been defined in McCallum et al.^31^ that is recognised by a large number of NTD-targeting nAbs. It includes the Spike regions: 14-20, 140-158 and 245-264. Multiple other NTD mutations have been reported to affect neutralization of NTD-targeting nAbs. These NTD mutations include^32^ A67V, del69-70, T95I, G142D, del143-145, N211I, del212, and ins214 EPE. In ref.^33^, NTD mutations with strong (del144, R246A), moderate (L18F, T19A, H164Y, D253G, D253Y), and mild (D80A, N149Q, S252F) effect on antibody neutralization were described. This data was collectively used in the overlap analysis of Spike NTD mutations (Fig. 4).

### Acquisition of SARS-CoV-2 CD8^+^ and CD4^+^ T cell epitopes

We obtained SARS-CoV-2 CD8^+^ and CD4^+^ T cell epitope data from the dashboard reported by us^51^ (https://www.mckayspcb.com/SARS2TcellEpitopes/; accessed on 15 May 2022) and the Immune Epitope Database (IEDB)^52^ (https://www.iedb.org; accessed on 15 May 2022) by querying for the organism name: “SARS-CoV2” (taxonomy ID: 2697049), host: “human”, and assay: “T cell positive”. The compiled data was processed to only include epitopes with lengths between 9-11 residues for CD8^+^ and 13-20 residues for CD4^+^, which represent the typical range of HLA class I and II epitopes. Removing the epitopes with no or incomplete HLA allele information resulted in a total of 1,324 unique CD8^+^ and 961 unique CD4^+^ epitope-HLA pairs (Supplementary Fig. 6). The analysis in Fig. 5A is based on this complete set of known SARS-CoV-2 T cell epitopes.

For the analysis focused on epitopes targeted by T cells in the HK population (Fig. 5B), we determined class I and class II HLA alleles prevalent in HK. For class I alleles, we employed the IEDB’s “Population Coverage” tool (http://tools.iedb.org/population/) to identify 12 HLA class I alleles that together cover >99% of the HK population (Supplementary Fig. 10A, left panel). A total of 630 unique SARS-CoV-2 CD8^+^ T cell epitopes were associated with these alleles (Supplementary Fig. 10A, right panel). For class II alleles, we employed the Allele Frequency Net Database^53^ (http://www.allelefrequencies.net; accessed on 15 May 2022) and identified 13 HLA class II alleles that have an individual estimated population coverage of >5% in the HK population (Supplementary Fig. 10B, left panel). A total of 258 unique SARS-CoV-2 CD4^+^ T cell epitopes were associated with these alleles (Supplementary Fig. 10B, right panel).

### Overlapping T cell epitopes per mutation

To study whether the within-host mutations (minor allele variants) affect the T cell response generated against different SARS-CoV-2 lineages and under different vaccination status, we used the metric *Overlapping T cell epitopes per mutation*. It is computed as the number of T cell epitopes overlapping the within-host mutations observed in each group divided by the total number of within-host mutations observed in that group. The T cell epitope data used in the calculation of this metric was either from the complete set (Fig. 5A) or from the set specific to the HK population (Fig. 5B).

### Statistical analysis

For bootstrapping analysis, the measurement can be taken from the same sample measured repeatedly. For the other tests (e.g., Wilcoxon tests), the measurements were taken from distinct samples. All the statistical tests in this study are two-sided and no adjustment for multiple comparisons was performed unless specified.

## Supplementary Material

1

## Data Availability

The sequencing data used in this study can be access through NCBI Sequence Read Archive (SRA) with accession ID: XXX. The anonymised metadata are deposited at https://github.com/Leo-Poon-Lab/mutations-under-sarscov2-vaccination/XXX.
