# Supplementary material for: Within-host diversity of SARS-CoV-2 lineages and effect of vaccination
Source: Res Sq. 2022 Aug 11:rs.3.rs-1927944. Preprint. [Version 1] doi: 10.21203/rs.3.rs-1927944/v1 (PMC9387541; doi:10.21203/rs.3.rs-1927944/v1)
Supplement: 1 [file NIHPPrs1927944v1-supplement-1.pdf]

Supplementary Tables

Table 1. iSNVs in different regions.

Gene	Mean iSNVs per Kb	Mean iSNVs per Kb (adjusted)
Full genome	0.04535019	-1.37E-18
ORF1ab	0.04392511	3.43E-18
S	0.06269981	-5.83E-18
ORF3a	0.03001364	-6.78E-18
E	0.14527307	-1.29E-17
M	0.03996223	3.60E-18
ORF6	0.05237549	-2.34E-17
ORF7a	0.0324728	1.28E-17
ORF7b	0.02319486	-1.52E-17
ORF8	0.02135847	7.58E-18
N	0.04786782	-3.81E-18
ORF10	0.03746862	-1.68E-17

Table 2. Distribution of high-frequent iSNVs in different viral lineage and vaccination groups.

Lineage	Vaccine	Gene	Mutation (amino acid)	Proportion
B.1.1.63	Unvaccinated	ORF1ab	D5973N	0.28
B.1.36.27	Unvaccinated	ORF1ab	D5973N	0.26
B.1.36	Unvaccinated	ORF1ab	D5973N	0.15
Delta	Unvaccinated	ORF1ab	D5973N	0.13
Delta	Comirnaty	ORF1ab	D5973N	0.11
Alpha	Unvaccinated	ORF1ab	D5973N	0.04
Delta	CoronaVac	ORF1ab	D5973N	0.04
B.1.36.27	Unvaccinated	S	D843D	0.73
B.1.36	Unvaccinated	S	D843D	0.27
B.1.36.27	Unvaccinated	S	K811R	0.33
B.1.1.63	Unvaccinated	S	K811R	0.25
Alpha	Unvaccinated	S	K811R	0.21
Delta	Unvaccinated	S	K811R	0.17
Delta	Comirnaty	S	K811R	0.04
B.1.36.27	Unvaccinated	ORF1ab	S6096R	0.55
B.1.1.63	Unvaccinated	ORF1ab	S6096R	0.27
B.1.36	Unvaccinated	ORF1ab	S6096R	0.09
Delta	Comirnaty	ORF1ab	S6096R	0.05
Delta	CoronaVac	ORF1ab	S6096R	0.05
B.1.36.27	Unvaccinated	E	V75A	0.61
B.1.36	Unvaccinated	E	V75A	0.27
Delta	Unvaccinated	E	V75A	0.09
Alpha	Unvaccinated	E	V75A	0.03

Table 3. Differences in iSNVs between groups at full-genome level. Only pairs with significantly large difference ($p<0.05$ in Wilcoxon Rank Sum test and difference between median values >10%) are shown.

Variable 1	Variable 2	Median of variable 1	Median of variable 2	P value
**Number of iSNVs per Kb (adjusted)**				
Unvaccinated_B.1.1.63	Unvaccinated_Delta	-0.0218	0.0019	0.0002
Comirnaty_Delta	Comirnaty_Omicron	0.0328	-0.0023	0.0013
Unvaccinated_B.1.36.27	Unvaccinated_Delta	-0.0225	0.0019	0.0030
Unvaccinated_B.1.36	Unvaccinated_Delta	-0.0135	0.0019	0.0138
Comirnaty_Delta	Unvaccinated_Delta	0.0328	0.0019	0.0189
Unvaccinated_B.1.1.63	Unvaccinated_Omicron	-0.0218	-0.0007	0.0243
Unvaccinated_B.1.1.63	Unvaccinated_B.1.36	-0.0218	-0.0135	0.0379
**Minor allele frequency**				
NA				
**Nucleotide diversity (π)**				
Unvaccinated_B.1.36.27	Unvaccinated_B.1.1.63	7.22E-06	0	< 0.0001
Unvaccinated_B.1.1.63	Unvaccinated_Delta	0	1.97E-05	< 0.0001
Unvaccinated_B.1.36	Unvaccinated_B.1.1.63	5.24E-06	0	0.0002
Unvaccinated_B.1.1.63	Unvaccinated_Omicron	0	1.90E-05	0.0005
Unvaccinated_B.1.36	Unvaccinated_Delta	5.24E-06	1.97E-05	0.0096
Unvaccinated_B.1.1.63	Unvaccinated_Alpha	0	1.01E-05	0.0137
Unvaccinated_B.1.36.27	Unvaccinated_Delta	7.22E-06	1.97E-05	0.0165

Table 4. Synonymous and nonsynonymous nucleotide diversity on full genome and spike gene of different groups.

Gene	Group	π_N ($\pm SD$) (10^{-5})	π_S ($\pm SD$) (10^{-5})	$\pi_N - \pi_S$ (10^{-5})	π_N/π_S
Full genome	Combined (N=2053)	1.51 (1.43 ~ 1.59)	2.69 (2.32 ~ 3.06)	-1.18	0.56
Full genome	Comirnaty Delta (N = 58)	1.85 (1.57 ~ 2.13)	3.47 (2.81 ~ 4.12)	-1.62	0.53
Full genome	Comirnaty Omicron (N = 68)	1.95 (1.7 ~ 2.21)	3.06 (2.56 ~ 3.56)	-1.10	0.64
Full genome	Unvaccinated Alpha (N = 48)	1.64 (1.36 ~ 1.93)	1.25 (0.89 ~ 1.6)	0.40	1.32
Full genome	Unvaccinated B.1.1.63 (N = 805)	1.24 (1.16 ~ 1.32)	2.04 (1.87 ~ 2.22)	-0.81	0.61
Full genome	Unvaccinated B.1.36 (N = 221)	1.16 (1.02 ~ 1.29)	3.09 (2.19 ~ 3.98)	-1.93	0.38
Full genome	Unvaccinated B.1.36.27 (N = 707)	1.66 (1.55 ~ 1.77)	3.07 (2.35 ~ 3.8)	-1.41	0.54
Full genome	Unvaccinated Delta (N = 70)	1.97 (1.7 ~ 2.23)	2.90 (2.37 ~ 3.43)	-0.93	0.68
Full genome	Unvaccinated Omicron (N = 30)	1.67 (1.32 ~ 2.02)	2.79 (2.06 ~ 3.53)	-1.12	0.60
Full genome	CoronaVac Delta (N = 14)	1.61 (1.16 ~ 2.06)	3.11 (2.04 ~ 4.18)	-1.50	0.52
Full genome	CoronaVac Omicron (N = 32)	1.74 (1.4 ~ 2.08)	2.56 (1.89 ~ 3.24)	-0.82	0.68
S	Combined (N=2053)	1.93 (1.72 ~ 2.14)	4.92 (2.34 ~ 7.51)	-2.99	0.39
S	Comirnaty Delta (N = 58)	2.49 (1.81 ~ 3.16)	2.39 (1.37 ~ 3.41)	0.09	1.04
S	Comirnaty Omicron (N = 68)	2.62 (1.84 ~ 3.4)	6.56 (4.49 ~ 8.63)	-3.94	0.40
S	Unvaccinated Alpha (N = 48)	4.66 (3.16 ~ 6.15)	0.61 (0 ~ 1.22)	4.05	7.64
S	Unvaccinated B.1.1.63 (N = 805)	1.41 (1.17 ~ 1.65)	1.66 (1.29 ~ 2.03)	-0.25	0.85
S	Unvaccinated B.1.36 (N = 221)	1.84 (1.46 ~ 2.22)	9.84 (3.33 ~ 16.3)	-8.00	0.19
S	Unvaccinated B.1.36.27 (N = 707)	2.06 (1.78 ~ 2.33)	7.26 (2.04 ~ 12.5)	-5.21	0.28
S	Unvaccinated Delta (N = 70)	2.34 (1.45 ~ 3.24)	0.48 (0.13 ~ 0.82)	1.87	4.93
S	Unvaccinated Omicron (N = 30)	1.48 (0.71 ~ 2.24)	2.45 (0.68 ~ 4.23)	-0.97	0.60
S	CoronaVac Delta (N = 14)	0.81 (0.23 ~ 1.4)	3.39 (0.87 ~ 5.91)	-2.58	0.24
S	CoronaVac Omicron (N = 32)	1.8 (0.95 ~ 2.65)	7.91 (4.73 ~ 11.1)	-6.11	0.23

Table 5. Candidate genomic regions of positive selection within hosts.

Nucleotide range	Codon range	Gene region	Codons with nonsynonymous differences	P-value	π_N	π_S	π_N/π_S	Number of overlapping CD8 epitopes	P-value	Number of overlapping CD4 epitopes	P-value	Number of overlapping CD4 and CD8 epitopes	P-value
10172 to 10201	3303 to 3312 (40 to 49)	ORF1ab (nsp5)	3304, 3307, 3308, 3310, 3311, 3312	0.00	8.06E-05	0	-	1	0.6975	0	1	1	0.7557
4061 to 4252	1266 to 1329 (448 to 511)	ORF1ab (nsp3)	1272, 1281, 1282, 1287, 1291, 1293, 1295, 1297, 1299, 1301, 1306, 1311, 1313, 1314, 1316, 1318, 1319, 1321, 1323	0.01	5.21E-05	9.9E-06	5.26	8	0.3285	0	1	8	0.4449
2246 to 2278	661 to 671 (481 to 491)	ORF1ab (nsp2)	662, 664, 669, 671	0.05	3.8E-05	0	-	1	0.7138	0	1	1	0.7704
27520 to 27564	43 to 57	ORF7a	47, 50, 57	0.06	1.75E-05	0	-	5	0.0721	0	1	5	0.2793
20452 to 20457	6730 to 6731 (278 to 279)	ORF1ab (nsp15)	6731	0.11	2.63E-05	0	-	1	0.5125	0	1	1	0.5948
26362 to 26385	40 to 47	E	41, 45	0.13	3.76E-05	1.57E-05	2.39	3	0.6761	4	0.9718	7	0.8028
3683 to 3733	1153 to 1156 (335 to 336)	ORF1ab (nsp3)	1153, 1156	0.16	7.36E-06	0	-	2	0.2708	0	1	2	0.3804

The P-value for T cell epitope overlap is defined as the probability of observing at least the same number of overlapping epitopes in the gene's window of same length as the codon range.

Table 6. Number of samples with at least one detected iSNV and number of total analysed samples, stratified by virus lineages and vaccination status.

	Comirnaty	CoronaVac	Unvaccinated
**Alpha**	0	0	30/48
**Delta**	51/56	10/12	50/70
**Omicron**	51/57	24/26	21/24
**B.1.36**	0	0	123/220
**B.1.36.27**	0	0	403/697
**B.1.1.63**	0	0	354/760

Supplementary Figures

Figure 1. The sequencing depth in sliding window of 200bp of the samples included in this study. Each grey line is representative of one individual sample, and the red line shows the average of all samples. The dashed line showed depth of 100 reads.

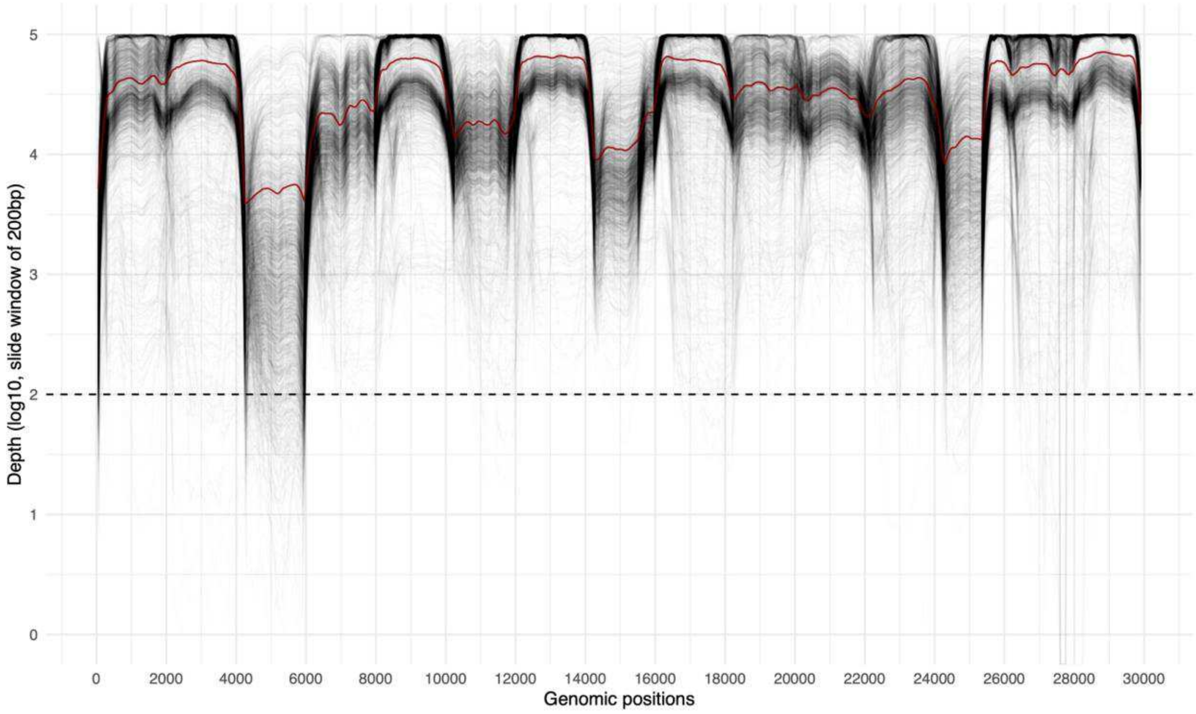

Figure 2. Correlation of Ct value between different factors. The regression lines are showed in blue. **(A)** Correlation between Ct value and number of iSNVs per Kb; **(B)** Correlation between Ct value and detection lag (time post symptom onset in days); **(C)** Correlation between detection lag and number of iSNVs per Kb; **(D)** Correlation between Ct value and minor allele frequency; **(E)** Correlation between Ct value and number of iSNVs per Kb (adjusted).

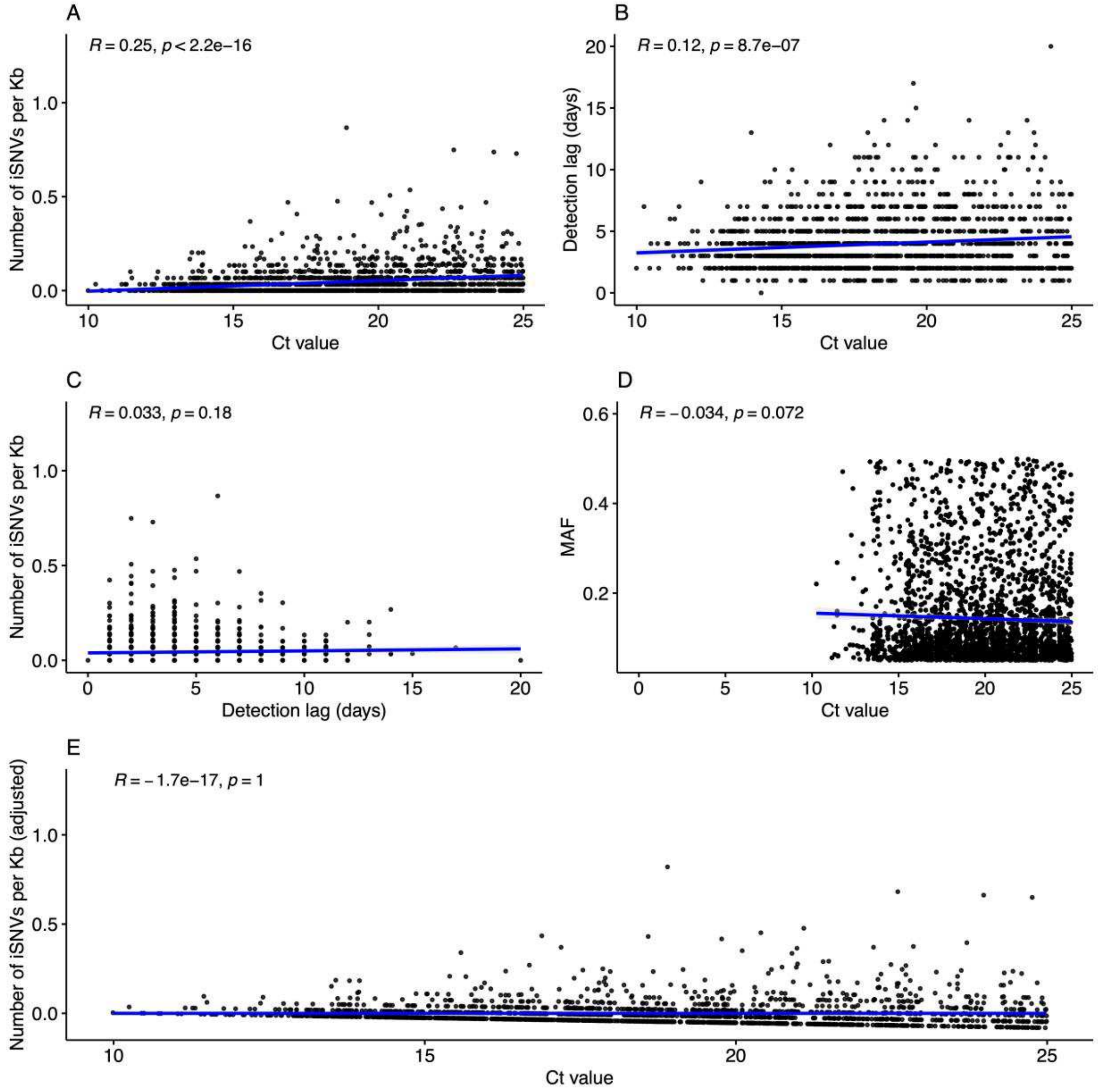

Figure 3. Within-host mutations profiles among different groups. Different vaccination statuses were compared, the data used here are the same as the data used in the Figure 2A-2C and Figure 3A-3C of the main text. Boxplots indicate median and inter-quartile ranges (IQR), and whiskers represent value ranges up to 1.5 * IQR. Pairwise comparisons within groups were tested by two-sided two-sample Wilcoxon tests, the pairs with P value ≤ 0.01 and ≤ 0.05 were labelled with "**" and "*" respectively. **(A)** Full-genome incidence of iSNVs (adjusted number of iSNVs per Kb) of different samples. **(B)** Full-genome abundance of iSNVs (minor allele frequencies) of different samples. **(C)** Full-genome nucleotide diversity (π) of different samples.

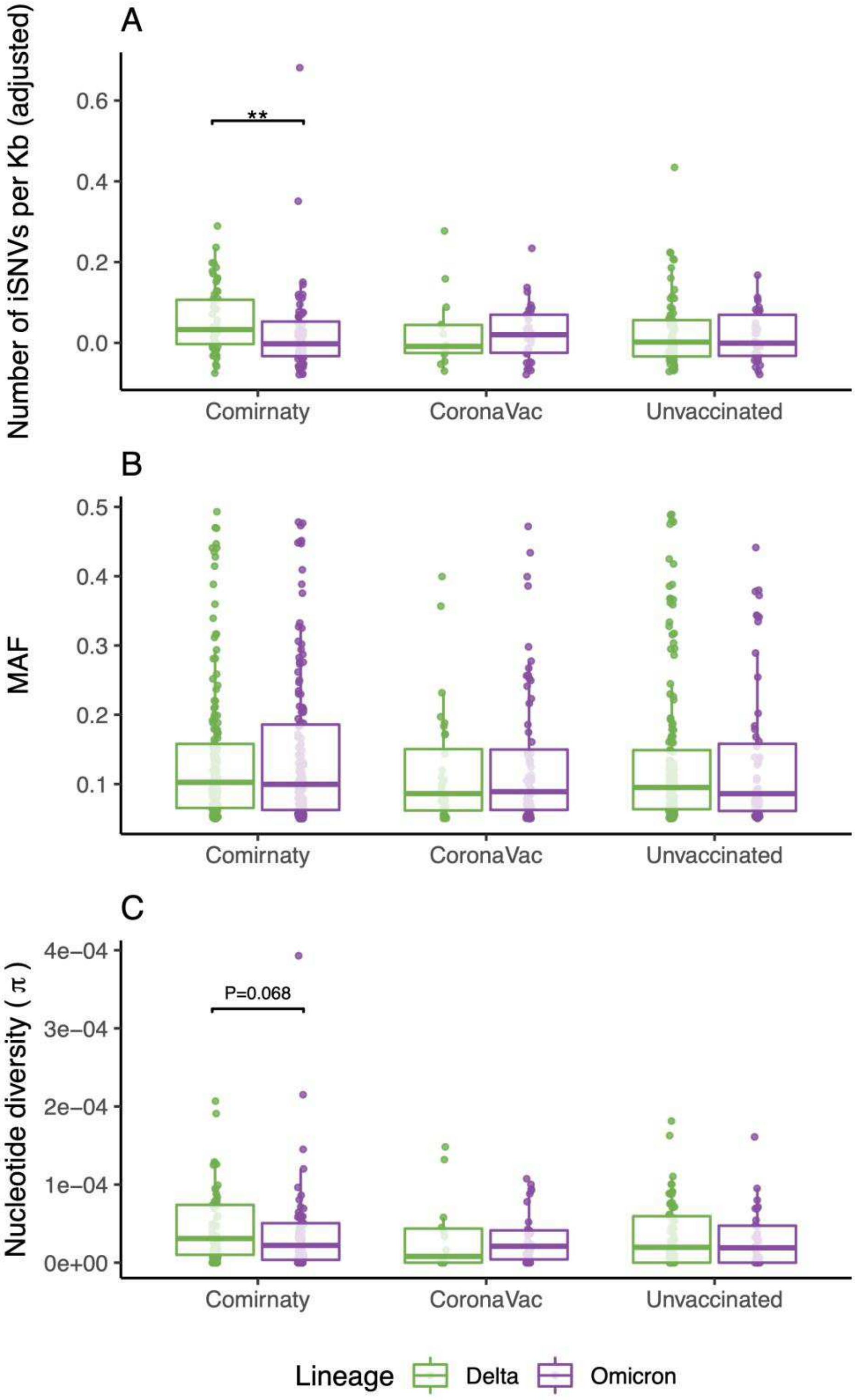

Figure 4. Distribution of time post last dose in the vaccinated samples.

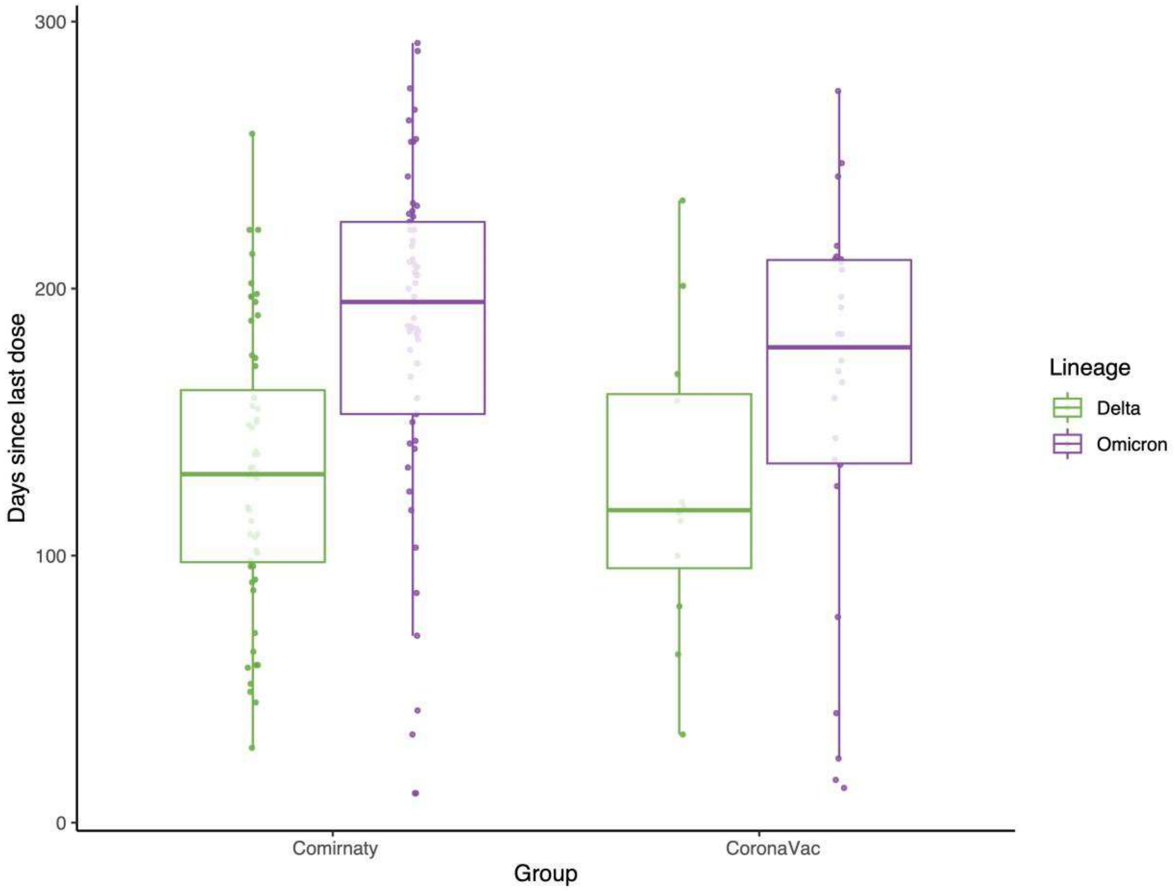

Figure 5. Sliding window analysis of Synonymous/Non-synonymous nucleotide diversity in different genes. Sliding windows size of thirty codons and step size of one codon were used because this did not exceed the length of ORF10 (thirty-nine codons).

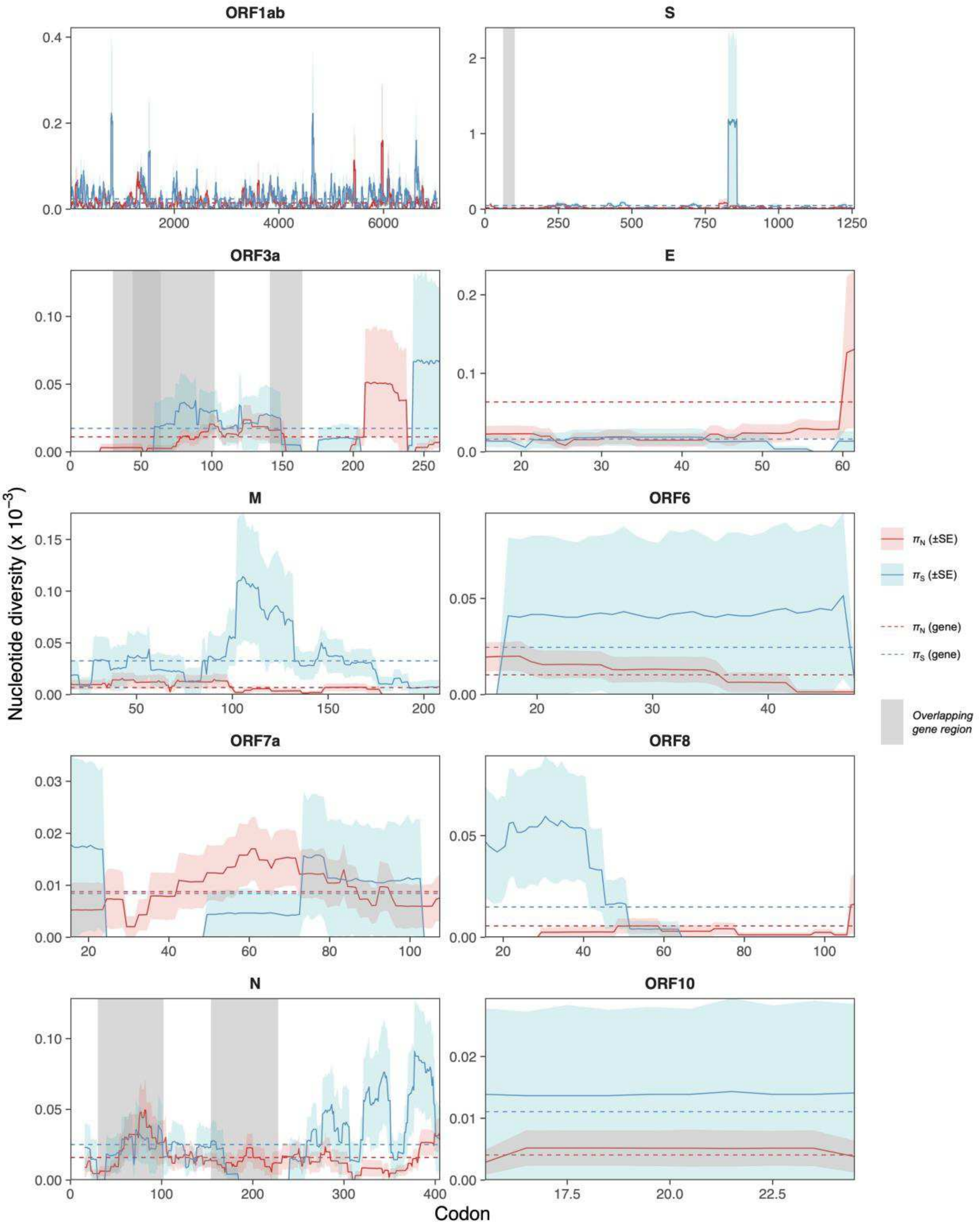

Figure 6. Antibody binding prediction of receptor-binding domain (RBD) mutations in vaccinated (A) Omicron and (B) Delta samples. The identified mutations in both Comirnaty and CoronaVac vaccinated samples were labelled in the plot (orange dots). The blue/grey lines show the total antibody binding before/after the mutations are introduced into the RBD region. The difference in y axis between orange and grey dots at the same amino acid site represents the loss of antibody binding under mutation, the differences in percentage were labelled in brackets. The calculations are based on deep mutational scanning of a large set of RBD targeting antibodies which are known to neutralize Wuhan-Hu-1. (https://jbloomlab.github.io/SARS2_RBD_Ab_escape_maps/escape-calc/).

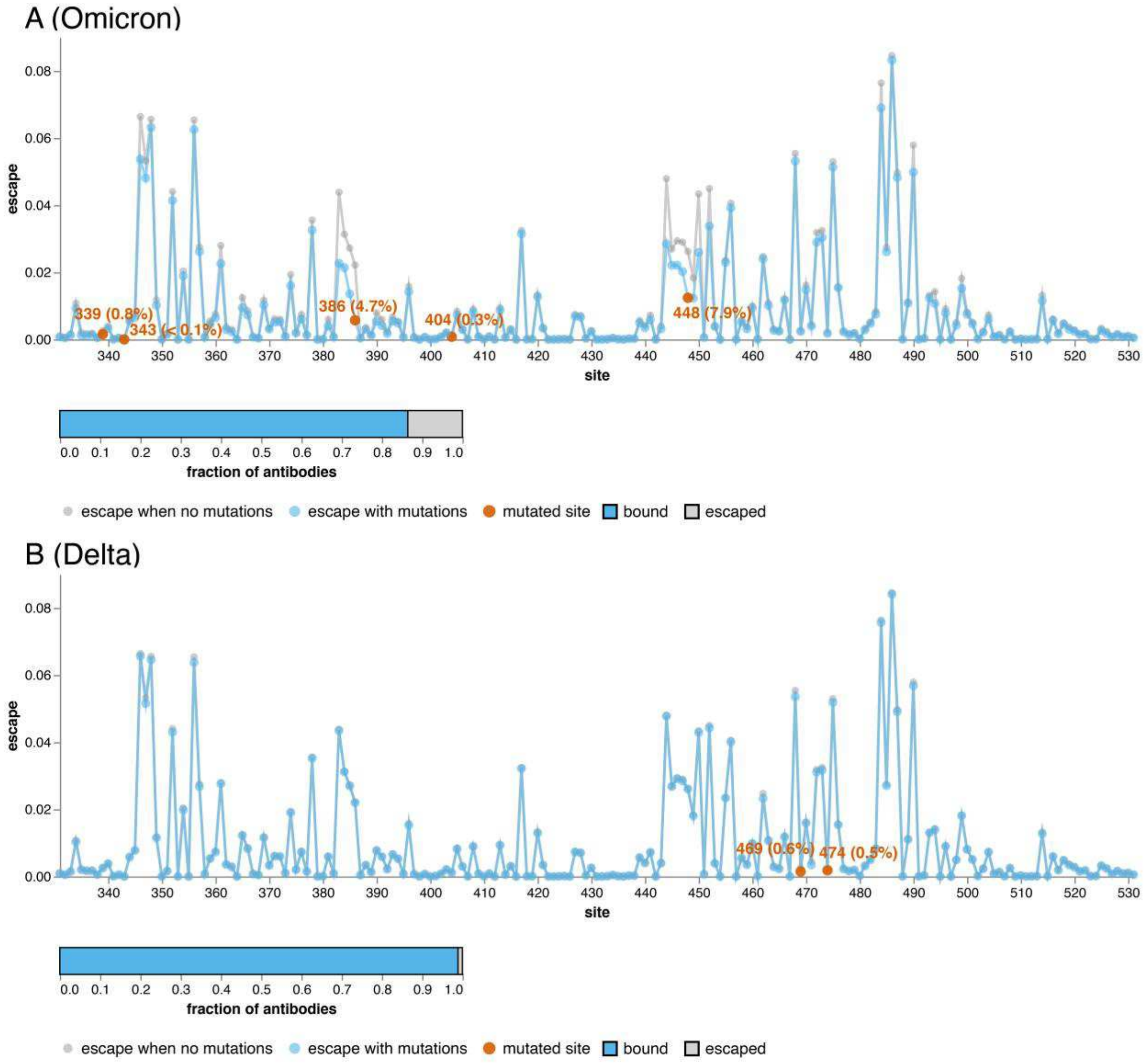

Figure 7. Distribution of CD4+/CD8+ T cell epitope-HLA pairs included in this study. (**A**) Distribution in different genomic regions; (**B**) Distribution in different HLA regions.

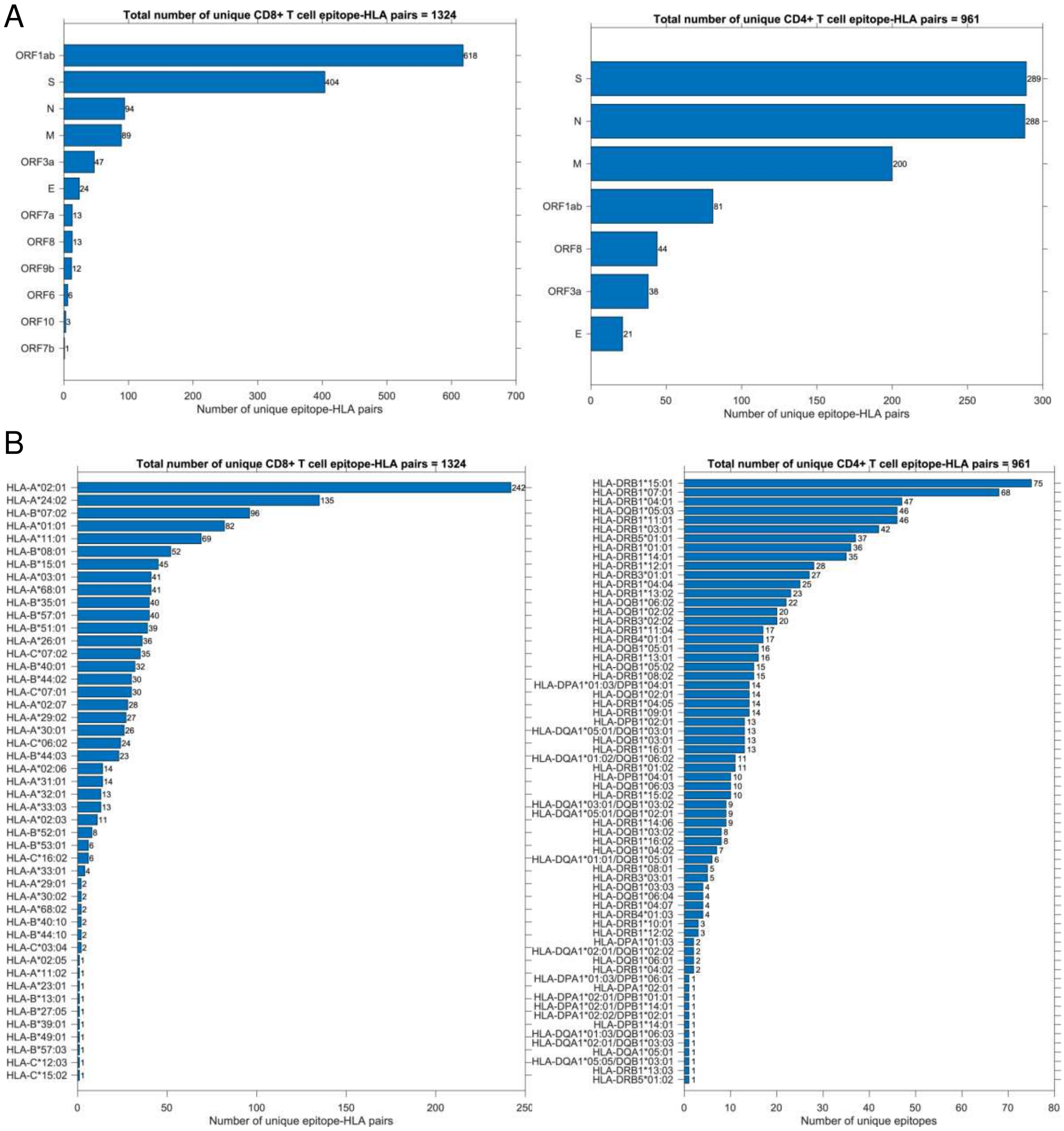

Figure 8. Average number of overlapping epitopes per mutation in different groups.

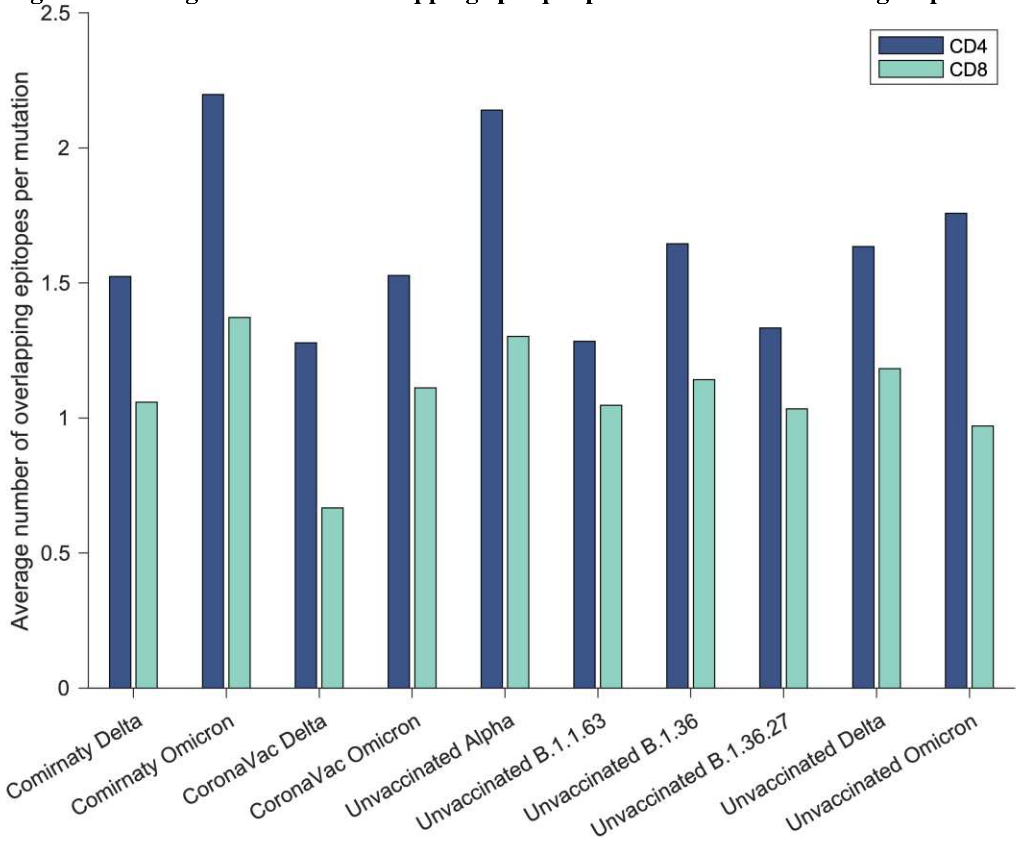

Figure 9. Overlapping of CD4+/CD8+ T cell epitopes per mutation in Spike between vaccinated and unvaccinated samples. (**A**) analysis based on unique T cell epitopes. (**B**) analysis based on epitope-HLA pairs specific to Hong Kong population.

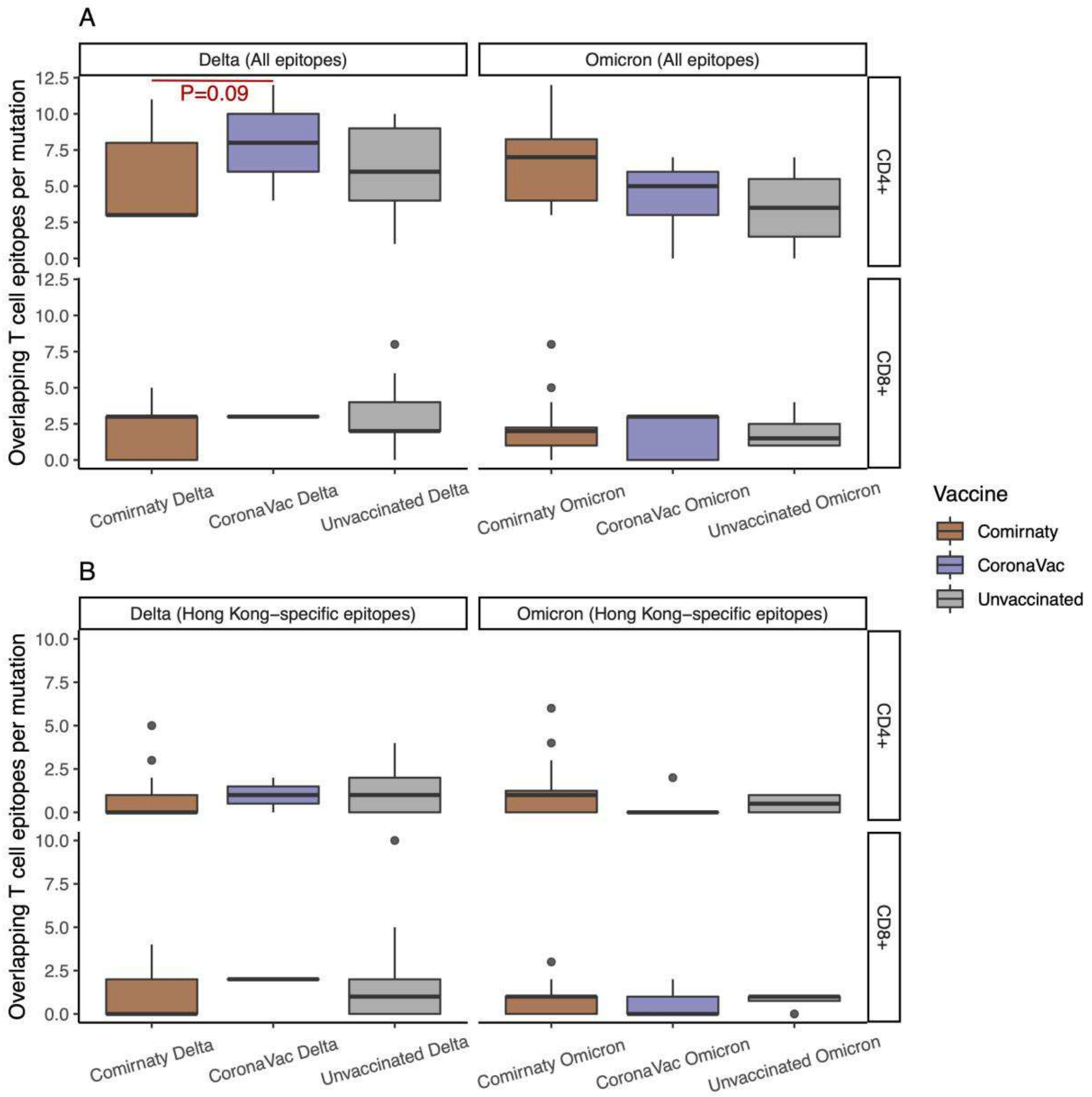

Figure 10. Distribution of CD4^{+}/CD8^{+} T cell epitope-HLA pairs specific to Hong Kong population

A

No.	HLA allele	Coverage in HK population (%)#
1	HLA-A*11:01	49.12
2	HLA-A*24:02	28.4
3	HLA-B*40:01	28.01
4	HLA-A*02:07	24.46
5	HLA-A*33:03	18.98
6	HLA-A*02:03	14.98
7	HLA-B*13:01	14.95
8	HLA-A*02:01	12
9	HLA-A*02:06	9.17
10	HLA-B*51:01	8.39
11	HLA-A*11:02	7.83
12	HLA-B*15:01	4.34

#Source: IEDB

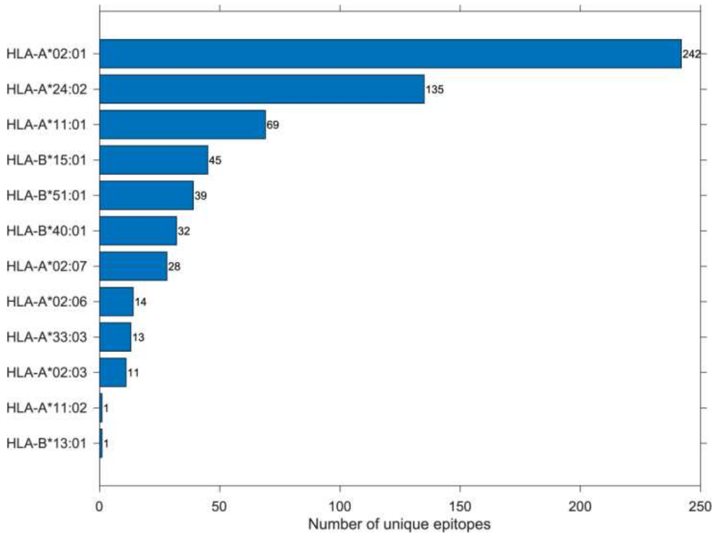

B

No.	HLA allele	Coverage in HK population (%)#
1	HLA-DQB1*03:01	23.55
2	HLA-DQB1*03:03	15.75
3	HLA-DRB1*09:01	15.37
4	HLA-DRB1*12:02	14.39
5	HLA-DQB1*05:02	11.76
6	HLA-DQB1*06:01	11.27
7	HLA-DRB1*15:01	9.65
8	HLA-DRB1*03:01	6.79
9	HLA-DQB1*02:01	6.69
10	HLA-DQB1*03:02	6.29
11	HLA-DRB1*04:05	6.21
12	HLA-DRB1*08:03	5.94
13	HLA-DRB1*11:01	5.3

#Source: AFND (Population coverage > 5%)

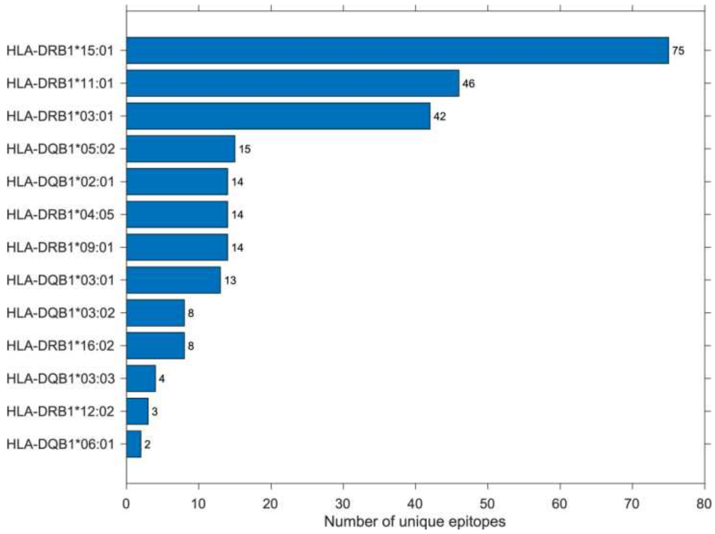

Supplementary Files

This is a list of supplementary files associated with this preprint. Click to download.

- reportingsummary.pdf
- checklist.pdf
